# Characterization of Oestrus Cycles in Namibian Swakara and Damara Sheep through Determination of Circannual Plasma Progesterone Levels

**DOI:** 10.1155/2019/5320718

**Published:** 2019-07-11

**Authors:** Erick Kandiwa, Borden Mushonga, Oscar Madzingira, Alaster Samkange, Alec Bishi, Dolly Tuaandi

**Affiliations:** School of Veterinary Medicine, Faculty of Agriculture and Natural Resources, University of Namibia, P. Bag 13301, Pioneers Park, Windhoek, Namibia

## Abstract

A year-long prospective study characterized the seasonality of oestrus cycles in primiparous, nonpregnant Swakara (n=8) and Damara (n=5) ewes through surveillance of plasma progesterone (P4) levels. During this period, Swakara and Damara groups evidently averaged 23 oestrus cycles with an average length of 17 days. Damara ewes showed greater mean peak plasma P4 levels (11.4±0.16ng/ml) than Swakara ewes (5.4±0.11ng/ml) (*P*<0.05). Oestrus cycles in Damara ewes showed relatively uniform plasma P4 peaks throughout the year ranging from 10.6±0.16 to 12.6±0.24ng/ml. In Swakara ewes, P4 peaks were highest in the autumn oestrus cycles (from 7.1±0.16 to 7.5±0.11ng/ml), rapidly declining through winter to 2.2±0.08ng/ml by midspring and then rapidly increasing to 4.9±0.37ng/ml at the commencement of summer, followed by a gradual increase from 5.7± to 7.1±ng/ml by the start of autumn. The annual mean area under the curve temporal progesterone measurements (AUCPM) in Damara ewes (115.9±18.6ng⁎day/ml) was greater than that in Swakara ewes (58.6±25.3ng⁎day/ml) (p<0.05). For Swakara ewes, the mean AUCPM in summer and autumn cycles (68.2±14.7 and 79.5±10.0ng⁎day/ml, respectively) were greater than those in spring and winter cycles (28.7±12.3 and 55.0±27.3ng⁎day/ml), respectively (*P*<0.05). There was no seasonal variation in the exposure of the Damara ewes to P4 in between seasons (*P*>0.05), though, however, the Damara ewes had greater P4 levels than the Swakara ewes (*P*<0.05). Progesterone profiles showed that Swakara ewes possessed ‘residual' seasonality, whereas the Damara ewes were no longer seasonal. The implications of this disparity in the seasonal exposure of Swakara and Damara ewes to luteal P4 on fertility warrant further investigation.

## 1. Introduction

Sheep production represents about 57% of small stock production in Namibia. The Dorper is the main commercial breed, with smaller contributions from the Swakara, Damara, and Van Rooy breeds [1]. The Swakara sheep (formerly Karakul), a fat-tailed medium-sized breed of sheep, has been bred in Namibia since 1907 when it was imported by sea from Bukhara, Asia. This breed has adapted to the arid semidesert conditions in southern Namibia and it is farmed primarily for lamb pelts that fetch niche prices on the world market, but also for meat, wool, and milk. Annual export earnings from Swakara pelts are estimated at 5 million euros [2]. The Swakara ewe reputedly has an extended breeding season with the ability to lamb three times every two years. Swakara rams can also be successfully used for mating throughout the year [3, 4]. The Damara sheep is a fat-tailed indigenous sheep breed originating from the Asiatic mouflon (*Ovis orientalis *or* O. gmelini*), that has over centuries tracked across Africa from Asia. Credited to its history is this breed's ability to lamb throughout the year and also to thrive in the arid and semidesert conditions of southern Namibia [5]. Damara sheep have high tolerance to seasonal weight loss, good resistance to local sheep diseases [5], and good mothering abilities [6].

Sheep are seasonally polyoestrus animals [7]. Autumn (March–June) and spring (September–December) are the two main sheep breeding seasons in southern Africa [8]. In autumn, sexual activity in ewes is at its peak and a high lambing and twinning percentage later results from this activity. In spring, ewes have lower sexual activity and a lower lambing percentage results from such diminished levels of sexual activity [8, 9]. Sheep in the temperate latitudes are more sensitive to changes in photoperiod than those in the tropics hence the emergence of sheep breeds in the latter that can breed all year round and others which have extended breeding seasons. The autumn breeding season is the true, spontaneous breeding season. Breeding at any other time of the year is only possible through hormone treatments or the male effect [10, 11].

Progesterone in the cycling ewe is secreted by the corpus luteum, which is a result of the luteinization of the ovulating follicle's remnant. As this corpus luteum develops, it produces increasing amounts of P4, followed by a rapid decline (beginning at day 14 of the oestrus cycle) in the event of luteolysis that is triggered by prostaglandin F2_*α*_ (PGF2_*α*_) secreted from the uterus in the nonpregnant ewe [12, 13]. The decrease in P4 at the end of the luteal phase and the rise in oestradiol (from the Graafian follicle) provides a perfect theatre for a surge of luteinizing hormones (LH), ovulation, and oestrus on day zero of the cycle [7, 14].

Progesterone suppresses uterine contractility and promotes cervical closure and endometrial gland growth through cell proliferation and angiogenesis as well as promoting an increase in secretory activity of the oviduct in the cycling ewe. This role of P4 is important in the establishment of pregnancy through the promotion of endometrial receptivity of the blastocyst and attachment of the trophoblast by suppression of deleterious maternal immune responses [15, 16]. Studies have used the area under curve of temporal progesterone measurements (AUCPM) on the implantation of embryos and thus clinical pregnancy in assisted reproductive technology (ART) in women [1, 17]. Other studies have also used the quantity of P4 to predict the probability of pregnancy in cattle [1, 18].

In spite of limited studies on the reproductive endocrinology of indigenous sheep breeds, there is need of evidence-based ways to improve the Swakara's and Damara's reproductive adaptation to the arid semidesert conditions of southern Namibia. The objectives of this study were to characterize the seasonality of Namibian Swakara and Damara oestrus cycles and to determine the exposure of the endometria of either breed to P4 as a measure of their receptivity to implantation by continuous surveillance of plasma P4 concentrations for a full year.

## 2. Materials and Methods

### 2.1. Study Area

The study was conducted at Neudamm farm (20°31'00”S and 17°15'00”E) in the Khomas region of Namibia. This area is characterized by savanna vegetation dominated by shrub-veld with temperatures ranging from 7°C to 33°C. The annual rainfall in the area ranges from 300 to 400mm. In this region, four seasons are experienced, namely, winter (21^st^ of June to the 20^th^ of September); spring (21^st^ of September to the 20^th^ of December); summer (21^st^ of December to the 20^th^ of March) and autumn (21^st^ of March to the 20^th^ of June). Circannual photoperiod variations in the study area are such that the longest day is 2.75 hours longer than the shortest day [19].

### 2.2. Study Animals

Two groups of mature (2.5 to 3 years of age) primiparous, nonpregnant Swakara (n=8,* M* = 41.2 kg;* SEM* = 0.86 kg) and Damara ewes (n=5,* M* = 46.2 kg;* SEM* = 1.44 kg) were used in this study. These two breeds of sheep were reared together under similar conditions at Neudamm farm, a University of Namibia teaching farm and research station. For purposes of the study, the ewes were physically separated from the rams, but kept in constant sight and within range of smell of intact rams throughout the year. The same rams were used throughout the study to limit the influence of novelty of male effect [11]. The animals were raised on natural pastures and provided with supplementary lucerne hay when pastures were depleted in winter. Water was provided* ad libitum*. Both groups were kept in good health and given up to date vaccinations, deworming and treatment against diseases and external parasites as necessary according to the University of Namibia animal welfare guidelines.

### 2.3. Blood Collection

Between August 2016 and August 2017, blood samples were collected from each of the ewes for plasma P4 assays at 3-4 day intervals for a period of one year. About 3ml of blood was collected aseptically by jugular venipuncture into 3.5 ml VACUETTE® tubes (K2E K2EDTAS) between 09H00 and 11H00 using 18G needles. Plasma was recovered by centrifugation at 2500rpm for 15 minutes within 30 minutes of sampling and stored frozen at -80°C until required for P4 testing. Weekly determinations of packed cell volume (PCV) were made to ascertain the absence of anaemia as a result of sampling or other unforeseen causes.

### 2.4. Determination of Plasma P4 Concentrations

The determination of P4 concentrations was done using direct competitive Enzyme-Linked Immunosorbent Assays commercial kits (ELISA–P4 ELISA, DRG diagnostics, DRG Instruments GmbH, Germany) as described by Anghel and coworkers [20]. Plasma samples and standard samples were placed in 96-well microtiter plates coated with rabbit anti-P4 polyclonal antibodies. A wash solution was then used to remove any unbound P4. P4 conjugated to horseradish peroxidase was then introduced to bind with any unoccupied antibodies. Subsequent introduction of the substrate, 3,3',5,5'-tetramethyl-benzidine diimine (TMB), and its digestion would cause a colour change (to blue) and a further colour change (to yellow) when the reaction was stopped by 0.5M sulphuric acid (H_2_SO_4_). The amount of colour change when measured at 440nm was inversely proportional to the amount of P4 in the plasma sample. The sensitivity of the P4 assay was 0.1ng/ml.

### 2.5. Data Analysis

Using Microsoft Excel 2013, the standard solutions were used to create a standard 2^nd^ order polynomial curve (R^2^ ≥ 0.9) from which the samples' P4 concentrations were determined. P4 values were arranged in chronological order on an Excel spreadsheet. Arrayed P4 values for different ewes within each group were physically adjusted over time to allow the animals' oestrus cycles to ‘coincide' for computation of mean P4 values. Since it was not feasible for the researcher to visually appraise and determine the length of oestrus cycle during the study period, the design was to use the P4 levels to determine the end of each oestrus cycle. The sharp drop in the P4 concentration after a peak was used to mark the end of each oestrus cycle. Using these mean values, simple moving averages were generated using the Statistical Package for Social Sciences (SPSS) version 25 and used to determine and depict the trend of P4 levels over time. P4 curves were compared within groups and between groups over time. Peak P4 values from each cycle were compared between seasons within groups and between groups. The area under the curve temporal progesterone measurements (AUCPM) was calculated in SPSS as a measure of the ‘amount' of P4 each ewe was exposed to within each oestrus cycle. The AUCPMs were compared between seasons within groups and between groups. The two-way ANOVA for repeated measures was used to compare the P4 and AUCPM values. Tukey's HSD post hoc test was then used to determine the significance of the seasonal difference in P4 and AUCPM values between the Swakara and the Damara ewes. The coefficient of variation (CV) was determined for each seasonal mean value of P4 and AUCPM. P values ≤0.05 were considered significant.

## 3. Results

A total of 23 oestrus cycles each were encountered in both the Swakara and Damara ewes during the study period of 366 days. The average cycle length was 17 days.

**Figure 1:**
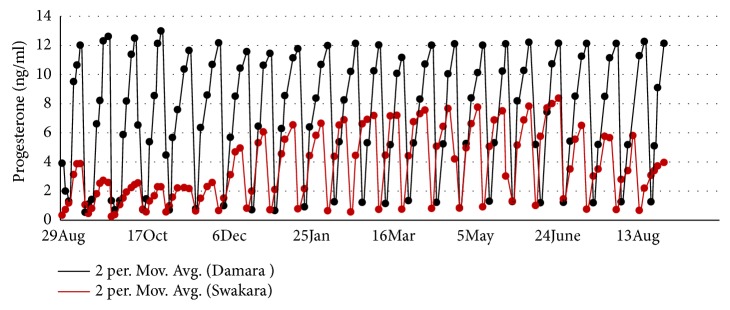
Mean plasma P4 levels in nonbred Swakara (n=8) and Damara ewes (n=5).

The Swakara ewe oestrus cycles had the greatest plasma P4 peak (8.2±0.12ng/ml) at the commencement of winter (20^th^ June) which, however, rapidly decreased to the lowest peak (2.2±0.08ng/ml) in mid-spring (October) (Figure 1). The P4 peaks of the oestrus cycles then rapidly increased to 4.9±0.37ng/ml at the commencement of summer (21^st^ December). There was a gradual increase in the P4 peaks of summer oestrus cycles from 5.7±0.17 to 7.1±0.16ng/ml at the commencement of autumn (20^th^ March). The P4 peaks in the autumn oestrus cycles were relatively constant (ranging from 7.1±0.16 to 7.5±0.11ng/ml) and only reached maximum (8.2ng/ml) around the 20^th^ of June. The oestrus cycles in the Damara ewes were relatively constant with plasma P4 peaks ranging from 10.6±0.16 to 12.6±0.24ng/ml.

Swakara ewe oestrus cycles in winter had the lowest plasma levels of P4 (max 2.2+0.08ng/ml) which were significantly lower than P4 levels in Swakara summer oestrus cycles (*P*<0.05). The Damara ewe oestrus cycles throughout the year had significantly greater levels of plasma P4 levels than both the summer and winter Swakara oestrus cycles (*P*<0.05).

The overall mean seasonal P4 peaks oestrus cycles of the Damara ewes were significantly greater than those in the Swakara ewes [*F*(6,24) = 64.68,* p*<0.001]. There was, however, no significant difference in the spring, summer, autumn, and winter mean P4 peak levels in the Damara ewes (*p*>0.05).

The summer and autumn oestrus cycles in Swakara ewes had significantly greater mean peaks of P4 (6.36±0.24ng/ml;* CV*=8.27% and 7.24±0.06ng/ml;* CV*=1.92%, respectively) than the spring and winter oestrus cycles (2.86±0.5ng/ml;* CV*=39.12% and 5.0±0.69ng/ml;* CV*=36.12%, respectively) (*P*<0.05) (Table 1). There was no significant difference between the Swakara summer mean P4 and the Swakara autumn mean P4 levels (*p*>0.05). The Swakara winter mean P4 level was, however, significantly greater than the Swakara spring mean P4 level (*p*<0.05).

The overall mean seasonal AUCPM of the Damara ewes were significantly greater than those of the Swakara ewes [*F*(6,24) = 9.6,* p*<0.001]. There was, however, no significant difference in the mean AUCPM between seasons in the Damara ewes [*F*(2,14) = 0.06,* p*=0.93]. There was a significant difference in the mean AUCPM between seasons in the Swakara ewes [*F*(2,14) = 5.84,* p*<0.03]. Post hoc analysis showed that the AUCPM levels for Swakara ewes in summer (68.18±6.58ng*∗*day/ml P4;* CV*=10.12%) and autumn (79.53±4.11ng*∗*day/ml P4;* CV*= 12.65%) were significantly greater than those in winter (55.01±10.32ng*∗*day/ml P4;* CV*=49.66%) and spring (28.74±12.31ng*∗*day/ml P4;* CV*=42.82%) (*p*<0.05).

## 4. Discussion

Analysis of P4 measurements for about 12 months in this study showed that there were 23 oestrus cycles for the Damara and Swakara ewes. The magnitude of the P4 fluctuations observed in this study indicates that both groups were cyclic throughout the year. The average length of the oestrus cycle of 17 days was in agreement with other studies and this is a reflection of the consistency of the oestrus cycle length in sheep [7]. Overall, the Damara ewes had significantly higher levels of P4 than the Swakara though being reared in the same photoperiod environment.

Three levels of plasma mean peak P4 concentrations emerged from this study; the breeding season mean peak P4 levels for Swakara ewes (6.4-7.2ng/ml); the ‘off-season' Swakara peak P4 levels (2.2-5.0ng/ml), and circannual Damara peak P4 levels (11.4ng/ml). These P4 are within the normal ranges encountered elsewhere worldwide. Peak P4 levels in Karakul ewes from temperate regions (Asia) during the breeding season ranged from 7.2- 15.2 ng/ml [21]. Peak P4 levels in Gaddi ewes in the temperate latitudes of India varied from 1.44 ng/ml (anoestrus) and 1.57 ng/ml (nonpregnant during the breeding season) to 5.16 ng/ml (pregnant), thus confirming that it is not unusual for P4 levels to vary widely between different breeds of sheep [22]. Peak P4 levels in Rideau Arcott x Polled Dorset during the breeding season of the temperate latitudes of Canada ranged between 4 and 6 ng/ml [7]. Peak P4 levels in Santa Ines ewes during the breeding season in subtropical latitudes ranged between 3.9 and 5.1 ng/ml [23].

The uniformity of the peaks of P4 observed in Damara sheep P4 measurements suggests that the Damara sheep in this study were nonseasonal and were probably influenced by the male factor [10, 11]. The farm manager indeed confirmed year round successful breeding with this breed at Neudamm farm (Beukes, personal communication). Provided that proper nutritional conditions and body conditions are well maintained, this nonseasonal nature of the Damara sheep can be manipulated to lamb three times in 2 years, representing a 50% increase in productivity. It is not uncommon for sheep breeds to lose seasonality under different photoperiodic conditions as observed with the St. Croix and the Barbados Blackbelly that are nonseasonal breeders under Tropical Caribbean photoperiod conditions but are still seasonal breeders under temperate American photoperiod conditions [24]. This phenomenon has also been observed with the Garut sheep breed of Indonesia [25].

In Swakara ewes, clear seasonal variations in P4 levels were observed. Oestrus cycles with lower P4 peaks but not complete anoestrus were observed in the Swakara ewes between September and November (the Namibian spring–summer seasons) which reveals memory of past seasonality in this breed, though it is quite clear that the nonbreeding period is somewhat reduced. This phenomenon means that the Swakara have an extended breeding season since up to 78% of their oestrus cycles displayed ‘normal' P4 peaks as compared to the anoestrus that would be expected in the nonbreeding season (up to 50% of the year) in full-blown seasonal breeders. A similar phenomenon was observed in experimental out-of-season (OOS) ewes at Virginia Tech whereby anoestrus was evident in the presence of diminished but discernible P4 peaks averaging 2-4 ng/ml [26]. These diminished P4 peaks experienced in spring could be due to insufficient luteinization and/or short lived corpora lutea [7]. The presence of an extended breeding season for the Swakara ewe was further supported by the farm manager's revelation that there are two breeding seasons (in late summer and early winter) for the Swakara at Neudamm farm (Beukes, personal communication).

The AUCPM of Damara ewes in spring (119.2±12.1ng*∗*day/ml), summer (122±19.4ng*∗*day/ml), autumn (114±17ng*∗*day/ml), and winter (110±24.2ng*∗*day/ml), showed relatively little variation throughout the study period. This finding further supports the suggestion that the Damara ewes at Neudamm farm were nonseasonal and can be bred all year round. The AUCPM of Swakara ewes in spring (28±12.3ng*∗*day/ml) was the lowest in comparison to the rest of the seasons, thus supporting the suggestion that Swakara ewes at Neudamm are still not able to breed in spring (a documented nonbreeding season in the southern hemisphere) [27]. The AUCPM of Swakara ewes in autumn, summer, and winter (79.5±10.1, 68.2±14.7 and 55.0±27.3ng*∗*day/ml, respectively) were significantly greater than those in spring. This finding supports the existence of an extended breeding season (incorporating autumn, summer, and winter) for the Swakara as further evidenced by the current ‘double' breeding of ewes per given year at Neudamm. Since the male was continuously present for both female breeds, their role in influencing reproductive endocrinology in the Damara and Swakara is difficult to apportion. Since the role of P4 in cycling ewes is to make the endometria receptive for implantation of the conceptus, it is reasonable to assume that breeding the Swakara ewes in autumn, summer and winter can be fairly successful since the endometrium is exposed to fairly high levels of P4, unlike in spring. The Damara endometria, however, due to the uniformly high exposure to P4, may explain the year round breeding success [17, 28].

The findings of this study have managed to characterize the circannual oestrus cycles in Damara and Swakara ewes at Neudamm. The relative exposure of endometria to each breed has also been successfully shown. Further studies are therefore needed with breeding experiments to prove or disprove the findings and theories emanating from this study and to document the gestational P4 levels in the Damara and Swakara ewes at Neudamm farm.

## Figures and Tables

**Table 1:** Comparison of plasma mean seasonal peak P4 and AUCPM in nonbred Swakara and Damara ewes.

Statistic or measure	*Spring *		*Summer*		*Autumn*		*Winter*	
	Swakara	Damara	Swakara	Damara	Swakara	Damara	Swakara	Damara
Mean P4±SEM (ng/ml)	2,86±0,5	11,6±0,29	6,36±0,24	11,24±0,18	7,24±0,06	11,1±0,1	5±0,69	11,51±0,21

Mean AUCPM±SEM (ng*∗*day/ml P4)	28,74±12,31	119,24±5,4	68,18±6,58	122,08±19,37	79,53±4,11	114±7,12	55,01±10,32	110,87±9,18

Coefficient of variation of P4 peaks (%)	39,12	5,64	8,27	1,62	1,92	2,18	36,62	4,80

Coefficient of variation of AUCPM (%)	42,82	10,12	21,58	15,87	12,65	15,30	49,66	21,90

AUCPM = area under the curve P4 measurement.

## Data Availability

The full data which was used for this research is available from the authors upon request.

## Additional Points

*Animal Welfare. *All the animals used in this study were handled in compliance with the ARRIVE guidelines and in accordance with the UK Animals (Scientific Procedures) Act 1986 and associated guidelines and the EU Directive 2010/63/EU for animal experiments.
